# Heart Transplant Rejection: From Histological Diagnosis to Precision Medicine

**DOI:** 10.31083/RCM50555

**Published:** 2026-10-14

**Authors:** Andrea Marzullo, Cecilia Salzillo

**Affiliations:** ^1^Department of Precision and Regenerative Medicine and Ionian Area, Pathology Unit, University of Bari “Aldo Moro”, 70124 Bari, Italy

**Keywords:** heart transplantation, heart transplant rejection, endomyocardial biopsy, immunosuppression, biomarkers

## Abstract

Heart transplantation remains the treatment of choice for patients with end-stage heart failure who are unresponsive to medical therapy or ventricular assist devices. Despite significant advances in surgical techniques and immunosuppressive protocols, heart transplant rejection (HTR) remains a major cause of morbidity and mortality among recipients. This narrative review summarizes current knowledge on HTR, with particular emphasis on immunopathogenic mechanisms, diagnostic approaches, histological classification, and therapeutic strategies, including the most recent non-invasive monitoring techniques. A narrative review of recent literature was conducted, including key publications and the updated International Society for Heart and Lung Transplantation (ISHLT) guidelines on rejection mechanisms, histological grading systems, and therapeutic strategies. HTR occurs via hyperacute, acute cellular, antibody-mediated, and chronic mechanisms, each characterized by complex immunological interactions between donor and recipient. Endomyocardial biopsy (EMB) remains the diagnostic gold standard, while innovations such as gene expression profiling, donor-derived cell-free DNA (dd-cfDNA), and donor-specific antibody (DSA) testing are redefining rejection monitoring. Modern immunosuppressive therapies, particularly tacrolimus-based regimens and B-cell-targeted agents, have reduced the incidence of rejection and improved graft survival. Ongoing refinement of diagnostic techniques and personalized immunosuppressive strategies is essential to enhance post-transplant outcomes. A multidisciplinary approach remains crucial for optimal management of HTR.

## 1. Introduction

Heart transplantation represents the gold standard treatment for patients with acute or chronic heart failure who remain symptomatic or whose clinical condition progressively deteriorates despite optimal medical therapy [1,2,3].

In 2020, over 7000 heart transplants were performed worldwide, with approximately 90% concentrated in the United States and Western Europe [1]. The first human donor-to-recipient heart transplant was performed in 1967 by Dr. Christiaan Barnard; however, the patient died 18 days postoperatively due to a severe infection [4,5,6,7].

Since then, advances in surgical techniques, donor selection, organ preservation, prophylactic protocols, and immunosuppressive therapies have significantly improved the survival and quality of life of transplant recipients [3,8]. Notable innovations include the use of extended criteria donors, *ex vivo* organ perfusion, and more refined and personalized immunosuppressive strategies [3,9,10], which have reduced procedure-related complications such as infections and rejection, although rejection remains a major clinical challenge.

Heart transplant rejection (HTR) is one of the most feared complications, with an incidence of approximately 22.7% during the first post-transplant year among adult patients, according to the United Network for Organ Sharing (UNOS) registry [11,12]. The 2021 report from the Scientific Registry of Transplant Recipients (SRTR) indicates that the cumulative incidence of acute rejection at one year has remained relatively stable over the past decade, ranging from 18% to 32% depending on recipient age [13,14,15,16].

Rejection can occur within the first six months after surgery, a period of maximum immunological vulnerability, but remains a risk in subsequent years. The main forms include hyperacute rejection, acute cellular rejection (ACR), antibody-mediated rejection (AMR), and chronic rejection characterized by cardiac allograft vasculopathy (CAV) [14,15,16], each driven by distinct pathogenic mechanisms: hyperacute rejection is mediated by preformed antibodies against donor antigens, ACR by a T-cell response, AMR by recipient antibodies and complement activation, and chronic rejection by progressive graft damage and typical coronary artery disease [17,18,19].

The clinical challenge lies not only in timely recognition and treatment of rejection but also in preventing its long-term consequences, including graft deterioration and increased mortality [17,20]. Despite substantial advances in surgical techniques and immunosuppressive regimens, the clinical management of HTR remains highly heterogeneous across centers due to variations in surveillance strategies, interpretation of diagnostic tools, and therapeutic thresholds, reflecting the absence of universally standardized, evidence-based pathways, particularly for chronic and antibody-mediated rejection.

The aim of this review is not only to summarize current knowledge on heart transplant rejection but also to highlight the ongoing transition from conventional histopathological diagnosis towards a precision medicine approach. Particular emphasis is placed on integrating endomyocardial biopsy (EMB) with molecular profiling, donor-derived circulating cell-free DNA, gene expression profiling (GEP), advanced imaging techniques, and emerging artificial intelligence-based approaches. Focusing on the convergence of pathology, immunology, and noninvasive diagnostics, this review offers an updated perspective on how rejection surveillance and management can evolve beyond traditional biopsy-focused strategies.

## 2. Materials and Methods

This narrative review was conducted through a literature search using the PubMed, Embase, and Web of Science databases. The search strategy included combinations of the following keywords: “cardiac transplantation”, “acute cellular rejection”, “antibody-mediated rejection”, “endomyocardial biopsy”, “gene expression profiling”, “donor-derived cell-free DNA”, “cardiac allograft vasculopathy”, and “precision medicine”.

Articles were selected based on their relevance to the topic of heart transplant rejection and precision medicine. Priority was given to International Society for Heart and Lung Transplantation (ISHLT) guidelines, consensus statements, prospective studies, registry analyses, systematic reviews, and landmark clinical trials. References addressing the pathophysiology, diagnosis, monitoring, and treatment of acute cellular rejection, antibody-mediated rejection, and cardiac allograft vasculopathy were included. Additional studies were identified through manual screening of the bibliographies of relevant articles.

Case reports, articles with limited clinical relevance to heart transplant rejection, and studies not directly related to the scope of the review were considered lower priority.

## 3. Pathophysiology of Heart Transplant Rejection

The pathophysiology of HTR is driven by a complex and dynamic interaction between immune recognition mechanisms and tissue injury pathways. A clear understanding of these processes is essential for interpreting histological findings, integrating molecular biomarkers, and developing precision medicine approaches for personalized patient management.

### 3.1 Immune Recognition and Alloimmunity

The major histocompatibility complex (MHC), particularly human leukocyte antigen (HLA) is the core of allogeneic recognition and rejection response after transplantation. HLA molecules express graft peptides that are recognized as “non-self”, thereby promoting adaptive immune responses and the formation of donor-specific antibodies (DSAs) [21,22,23].

Allospecific antigen presentation occurs through direct, indirect, and, to a lesser extent, semi-direct pathways. In the direct pathway, donor antigen-presenting cells (APCs) express allogeneic MHC molecules that are directly recognized by recipient T cells. In the indirect pathway, donor-derived peptides are processed and presented by recipient APCs to CD4+ T lymphocytes. Both pathways contribute to different phases of rejection, specifically, the direct pathway dominates in the early phases, the indirect pathway is particularly relevant in late reactions and in memory formation [24,25,26].

The T lymphocytes (CD4+ helper and CD8+ cytotoxic) play a central role in mediating ACR by orchestrating the inflammatory response, the release of cytokines (IL-2, IFN-γ), and the activation of cytotoxic effectors [27,28]. At the same time, B cells and the antibodies they produce play a fundamental role in antibody-mediated rejection. Indeed, the production of DSAs can lead to endothelial injury, complement activation, and graft dysfunction [29]. Recent immunological and single-cell mapping studies have further elucidated the complex network of T and B cell subpopulations involved in cardiac rejection [30].

Once alloimmune recognition has occurred, downstream inflammatory and cytotoxic mechanisms lead to structural and functional damage of the cardiac allograft, which varies according to the type and timing of rejection.

### 3.2 Mechanisms of Injury

Hyperacute rejection is mediated by preformed antibodies, such as anti-ABO or anti-HLA antibodies, present in the recipient that immediately bind to the graft endothelium, causing microvascular thrombosis, extensive ischemia, and rapid loss of graft function. This phenomenon is now rare thanks to improved serological testing and matching [31].

The ACR is characterized by a T-cell response that results in a predominantly lymphocyte mononuclear infiltrate in the myocardium with evidence of myocyte damage. Pro-inflammatory cytokines and cellular activation lead to graft dysfunction if not treated promptly. Histopathological examination of EMB remains the reference for the diagnosis of ACR [32]. In addition to graft dysfunction, persistent myocardial inflammation during ACR may promote electrical instability and increase the risk of ventricular arrhythmias through myocyte injury and subsequent fibrotic remodeling.

In AMR, DSA bind to antigens presents on the coronary and microvascular endothelium, activating the complement cascade and recruiting effector cells such as macrophages. Complement cleavage products such as C4d/C3d deposited on the endothelial wall and the presence of intravascular macrophages, labeled by CD68, are useful immunohistochemical markers for the diagnosis of AMR on EMB. AMR is associated with a worse prognosis and an increased risk of developing allograft vasculopathy [33]. Microvascular injury and complement activation occurring during AMR may also contribute to myocardial ischemia and arrhythmogenic remodeling.

Chronic rejection manifests as a gradual process of endothelial damage, diffuse intimal hyperplasia, and progressive stenosis of the epicardial and intramyocardial arteries, and CAV is an accelerated form of allograft-specific coronary artery disease. Immunological mechanisms such as alloantibodies, repeated rejection episodes, immune cell-mediated inflammation, and non-immunological factors such as viral infections, particularly cytomegalovirus (CMV), dyslipidemia, hypertension, donor factors, act synergistically in the development of CAV [34,35]. Because CAV causes diffuse ischemic injury and progressive fibrosis, it represents an important substrate for ventricular arrhythmias and sudden cardiac death (SCD) in the long-term follow-up of heart transplant recipients.

Table 1 summarizes the main types of HTR, highlighting their underlying pathophysiological mechanisms and key diagnostic features.

**Table 1.** **Types of heart transplant rejection: pathophysiology and diagnostic features**.

Type of rejection	Pathophysiological and histological features	Key diagnostic markers	Typical timing
Hyperacute rejection	Preformed anti-ABO or anti-HLA antibodies cause immediate endothelial injury, complement activation, microvascular thrombosis, and ischemic necrosis.	C4d/C3d deposition; absence of T-cell infiltrates.	Minutes to hours after transplant.
Acute cellular rejection (ACR)	T-cell–mediated immune response with mononuclear infiltrates, myocyte damage, and interstitial edema.	CD3+, CD8+ lymphocytes; elevated troponin.	Mainly within first 6 months.
Antibody-mediated rejection (AMR)	Donor-specific antibodies bind endothelial antigens, activating complement and recruiting macrophages.	C4d+, CD68+ macrophages; circulating DSA.	Late acute or chronic.
Cardiac allograft vasculopathy (CAV)	Chronic immune-mediated endothelial injury with diffuse intimal hyperplasia and progressive vascular stenosis.	Coronary angiography, IVUS, OCT; inflammatory biomarkers.	Months to years post-transplant.

ABO, ABO blood group system; HLA, human leukocyte antigen; DSAs, donor-specific antibodies; IVUS, intravascular ultrasound; OCT, optical coherence tomography.

## 4. Diagnostic Approaches and Surveillance Strategies

Although EMB is the gold standard for diagnosis, modern rejection surveillance increasingly relies on integrating histopathological examination with molecular and noninvasive biomarkers. This shift represents a key element of precision medicine in cardiac transplantation.

### 4.1 Endomyocardial Biopsy: The Gold Standard

The EMB was introduced and developed for clinical use by the Stanford group and has progressively established itself as the gold standard for the diagnosis and surveillance of cardiac allograft rejection (Fig. 1) [36].

**Fig. 1.**
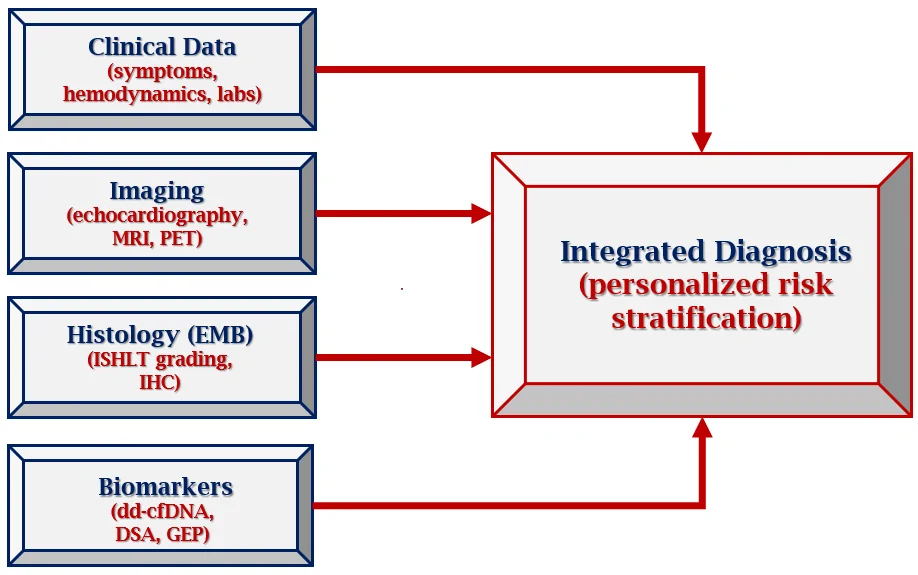
**Integrated diagnostic pathway for heart transplant rejection**. MRI, magnetic resonance imaging; PET, positron emission tomography; EMB, endomyocardial biopsy; ISHLT, International Society for Heart and Lung Transplantation; IHC, immunohistochemistry; DSA, donor-specific antibody; GEP, gene expression profiling.

The procedure involves the percutaneous collection of small myocardial tissue samples, usually obtained from the right ventricular septum using a flexible biopsy catheter. Transvenous access via the femoral or jugular vein is performed under fluoroscopic or ultrasound guidance; in selected cases, the left ventricle may be accessed through an arterial approach [37,38].

The recommended number of samples varies by center, but in current practice 3–5 pieces are commonly collected per procedure; many centers perform multiple biopsies in the first year post-transplant as part of standard surveillance protocols [39].

The EMB is a relatively safe technique, with rates of serious complications, such as tamponade and fatal arrhythmias, lower than 1% in most series, while overall complications reported in recent literature vary around 0.7–4% depending on the study and sampling site [40,41].

The clinical role of EMB remains central in the follow-up of transplant patients, allowing for the direct diagnosis of ACR and AMR, histological staging according to standardized criteria, and therapeutic guidance (escalation or de-escalation of immunosuppression). However, intrinsic limitations such as the invasive nature, sampling variability and relatively low sensitivity for focal lesions have been highlighted by recent reviews and multicenter studies [42,43].

Despite its central diagnostic role, EMB presents intrinsic limitations, including sampling error, interobserver variability, and reduced sensitivity for focal or early rejection. In the context of precision medicine, EMB should therefore be interpreted within an integrated diagnostic framework that combines histology with molecular biomarkers and clinical data, allowing for more accurate risk stratification and personalized therapeutic decisions.

### 4.2 Histological Grading Systems (ISHLT)

The first standardized classification scheme for cardiac rejection was proposed in 1990, subsequently, an ISHLT-led update was formally published in 2004 (revision published in 2005) in order to simplify and make the histological grades of ACR more prognostically relevant and to integrate emerging knowledge on AMR [44].

In the revised version, the classification of ACR is expressed with grades 0R–3R, specifically 0R = no rejection, 1R = mild, 2R = moderate, 3R = severe, while for AMR a scale has been adopted that integrates histological (H) and immunopathological (I) findings up to grade 3 for severe forms. These categories guide clinical decisions and therapeutic interventions [44].

It is important to distinguish rejection findings from benign or mimic entities, such as Quilty-like lesions, characterized by endocardial infiltrates predominantly of B lymphocytes that do not always correlate with clinically significant myocardial rejection. Correct identification of these lesions reduces the risk of false positives and unnecessary therapeutic approaches [45].

### 4.3 Emerging Non-Invasive Tools

In recent years, several non-invasive methods for monitoring rejection have emerged that can complement, and in some contexts reduce, the need for EMB, although they do not completely replace it (Fig. 2).

**Fig. 2.**
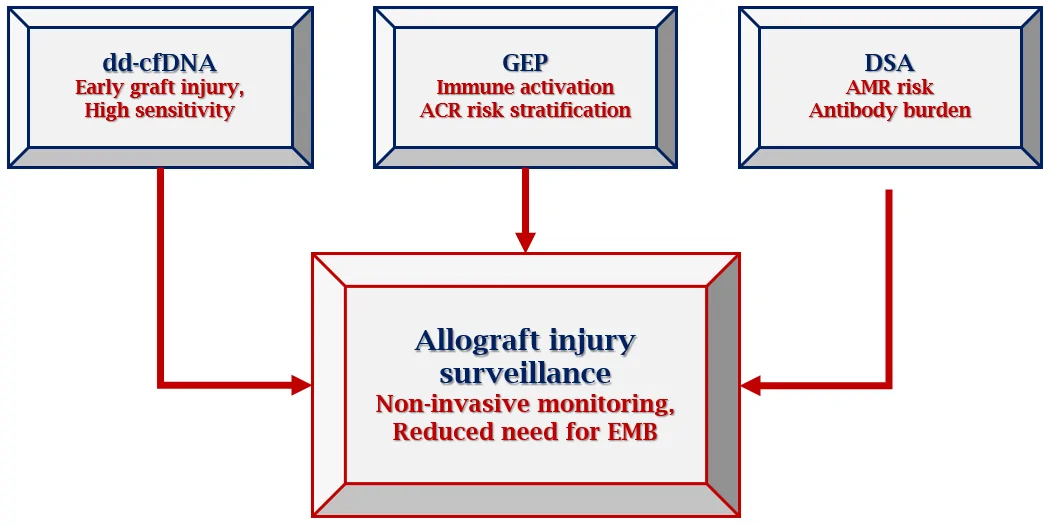
**Non-invasive biomarkers in heart transplant rejection**. GEP, gene expression profiling; DSA, donor-specific antibody; EMB, endomyocardial biopsy; dd-cfDNA, donor-derived cell-free DNA.

GEP is a peripheral blood gene expression test on peripheral leukocytes (e.g., AlloMap) that has been validated as screening tool to identify patients at low risk of clinically significant ACR and to reduce the number of surveillance biopsies in selected populations. Recent reviews have highlighted the clinical value of GEP but also the limitations related to infectious or inflammatory variables that can alter its performance [46,47].

However, GEP should be interpreted with caution, as its efficacy may be influenced by systemic inflammatory conditions, infections, and individual immune variability. Indeed, GEP is currently best suited to exclude clinically significant acute cellular rejection in stable patients, rather than as a definitive diagnostic tool.

Donor-derived cell-free DNA (dd-cfDNA) has emerged as a promising noninvasive biomarker of allograft injury. Mechanistically, dd-cfDNA reflects the release of fragmented donor DNA into the recipient’s circulation following damage to graft cells, thus representing a form of “liquid biopsy” capable of detecting immune-mediated damage before overt graft dysfunction develops. Several clinical studies have demonstrated its high sensitivity for the early diagnosis of both ACR and AMR, supporting its role in rejection surveillance and risk stratification [48,49,50].

However, the specificity of dd-cfDNA remains limited, as elevated levels can also occur in ischemic injury, infections, or procedural myocardial injury. Therefore, dd-cfDNA should not be considered a stand-alone diagnostic tool. Its optimal clinical application lies in an integrated approach that combines EMB, donor-specific antibody (DSA) assessment, and GEP, enabling more accurate diagnosis and personalized monitoring strategies in accordance with the principles of precision medicine.

Furthermore, from a precision medicine perspective, dd-cfDNA represents a form of liquid biopsy, enabling non-invasive detection of allograft injury and dynamic monitoring of immune-mediated damage. Its serial measurement may help reduce the number of surveillance biopsies in clinically stable patients when integrated with histological and immunological findings.

DSAs: monitoring anti-HLA DSA is essential for assessing the risk of AMR and subsequent allograft vasculopathy. Recent studies show that the appearance of de novo DSA, especially complement-binding ones, is associated with worse long-term outcomes [51,52].

Plasma biomarkers, such as high-sensitivity troponin, BNP/NT-proBNP: cardiac markers of damage and hemodynamic stress, have been studied as complementary tools to surveillance. High-sensitivity troponin and BNP/NT-proBNP can increase during rejection, but their specificity is limited by many cardiac and non-cardiac conditions. Multimodal combinations (dd-cfDNA + cardiac biomarker or GEP) appear to increase the diagnostic value compared to single tests [46,53].

The clinical utility of noninvasive biomarkers depends on their correct interpretation in specific clinical scenarios, as each tool has distinct strengths and limitations. GEP is primarily used in clinically stable patients during routine surveillance and is particularly useful for ruling out clinically significant ACR due to its high negative predictive value. However, its positive predictive value is limited, and abnormal results generally require confirmation with EMB.

dd-cfDNA is a sensitive marker of allograft damage and can detect rejection before overt graft dysfunction occurs. However, elevated levels are not specific for rejection and may also be observed in infection, ischemic injury, or other causes of myocardial damage.

DSA are primarily useful for identifying patients at increased risk of AMR and for long-term monitoring, although their presence does not necessarily indicate active rejection.

Conventional biomarkers, including troponin, BNP, and NT-proBNP, may reflect myocardial injury or ventricular stress, but lack sufficient specificity to distinguish rejection from other causes of graft dysfunction and therefore must be interpreted in conjunction with clinical, histologic, and immunologic findings.

Overall, no single biomarker can replace EMB. Rather, these tools are most effective when applied within a multimodal surveillance strategy that integrates EMB, DSA assessment, molecular profiling, and clinical assessment.

Echocardiography is the first-line noninvasive imaging technique for the routine monitoring of heart transplant patients and for the assessment of graft dysfunction. Conventional echocardiography provides information on ventricular dimensions, systolic and diastolic function, valvular alterations and possible pericardial complications [31,32].

In recent years, advanced echocardiographic techniques have significantly improved the ability to identify early subclinical myocardial dysfunction associated with rejection. Tissue Doppler Imaging (TDI) allows the assessment of regional myocardial velocities and alterations in diastolic function, while speckle-tracking echocardiography (STE) and Global Longitudinal Strain (GLS) analysis allow the detection of subtle mechanical alterations before changes in left ventricular ejection fraction become apparent [54,55].

Serial assessment using advanced echocardiographic techniques, particularly speckle-tracking echocardiography with global longitudinal strain analysis, may facilitate the earlier recognition of acute cellular rejection. Although echocardiography cannot replace histological evaluation, it represents a valuable complementary non-invasive tool for rejection surveillance in heart transplant recipients [54].

Non-invasive methods offer clear advantages such as less invasiveness, the possibility of serial monitoring, and have demonstrated useful capabilities in risk stratification and reducing unnecessary biopsies in selected populations. However, none of them currently replace EMB for the definitive diagnosis of ACR or AMR in the presence of clinical suspicion, since only biopsy allows direct morphological evaluation, the performance of immunohistochemical investigations (C4d, CD68) and the simultaneous exclusion of pathological mimics. Optimal clinical implementation appears to be based on an integrated approach that combines EMB with noninvasive tools, including circulating biomarkers, DSA monitoring, and advanced imaging techniques such as echocardiography, cardiac magnetic resonance imaging (MRI), and positron emission tomography (PET), in order to maximize sensitivity, specificity, and patient safety [32,46].

Table 2 summarizes the main diagnostic tools used in HTR, highlighting their clinical roles and limitations.

**Table 2.** **Diagnostic tools for heart transplant rejection: clinical role and limitations**.

Diagnostic method	Type	Clinical role and limitations
Endomyocardial biopsy (EMB)	Invasive	Gold standard for diagnosing ACR and AMR; allows histological grading and immunohistochemistry, but limited by invasiveness, sampling error, and interobserver variability.
Immunohistochemistry (C4d, CD68, CD3)	Invasive	Enhances diagnostic accuracy of EMB, particularly for AMR; dependent on tissue quality.
Gene Expression Profiling (GEP)	Non-invasive	Useful for excluding significant ACR in stable patients; influenced by infections and systemic inflammation.
Donor-derived cell-free DNA (dd-cfDNA)	Non-invasive	Sensitive marker of allograft injury; limited specificity for rejection when used alone.
Donor-Specific Antibody (DSA) Monitoring	Non-invasive	Identifies risk of AMR and CAV; presence does not always correlate with clinical dysfunction.
Advanced echocardiography (TDI, speckle-tracking, GLS)	Non-invasive	First-line method for transplant surveillance that allows for the early identification of subclinical myocardial dysfunction and supports the early diagnosis of rejection, although it requires histological confirmation in suspected cases.
Advanced imaging (T1/T2 mapping MRI, immunological PET)	Non-invasive	Detects myocardial edema, inflammation, and fibrosis; limited availability and standardization.
Plasma biomarkers (troponin, BNP, NT-proBNP)	Non-invasive	Easily accessible indicators of myocardial stress; low specificity for rejection.

TDI, Tissue Doppler Imaging; GLS, Global Longitudinal Strain; MRI, magnetic resonance imaging; PET, positron emission tomography; BNP, B-type natriuretic peptide.

## 5. Clinical Management of Rejection

Clinical management of HTR requires a structured and tailored approach based on rejection phenotype, severity, timing, and individual patient risk factors. Therapeutic strategies must balance effective immune suppression with the prevention of long-term toxicity.

### 5.1 Immunosuppressive Strategies

Immunosuppressive strategies are a cornerstone of post-heart transplant management, as the recipient’s immune system inherently recognizes the transplanted heart as foreign and may mount a rejection response. For this reason, long-term immunosuppressive therapy is required to modulate immune activation and to prevent both acute and chronic forms of rejection.

Current immunosuppressive strategies rely on the combined use of multiple drug classes that act on different stages of the immune response, thereby providing a synergistic effect while minimizing the need for high doses of individual agents. Corticosteroids exert broad anti-inflammatory and immunosuppressive effects; antimetabolites, such as azathioprine and mycophenolate mofetil, inhibit lymphocyte proliferation; calcineurin inhibitors, including cyclosporine and tacrolimus, suppress T-cell activation; and mTOR inhibitors (sirolimus and everolimus) interfere with intracellular signaling pathways involved in cell growth and immune cell expansion [31]. This multidrug approach has become the standard of care in heart transplantation.

Among calcineurin inhibitors, tacrolimus-based regimens have consistently been associated with a lower incidence of acute rejection compared with cyclosporine-based protocols. In addition, tacrolimus generally shows a more favorable tolerability profile in many clinical settings, contributing to improved short- and mid-term transplant outcomes. These findings have led to the widespread adoption of tacrolimus as the backbone of contemporary immunosuppressive strategies [56,57].

Despite the availability of standardized protocols, immunosuppressive strategies must be tailored to the individual patient. Comorbid conditions, potential drug-drug interactions, and the toxicity profiles of immunosuppressive agents play a crucial role in therapeutic decision-making. Over time, treatment adjustments may be required to balance adequate rejection prevention with the risk of complications related to long-term immunosuppression, such as infections, malignancies, and organ-specific toxicities. In this context, a personalized and dynamic approach to immunosuppressive management is essential to optimize graft survival and patient quality of life.

### 5.2 Treatment of Acute Rejection

The treatment of acute rejection after heart transplantation varies according to the underlying pathogenic mechanism, the severity of the clinical presentation, and the patient’s hemodynamic stability [58,59]. Early recognition and timely intervention are essential to preserve graft function and improve survival outcomes.

The standard therapeutic approach for moderate-to-severe ACR relies on high-dose corticosteroids, usually administered as intravenous boluses followed by gradual tapering. In steroid-resistant cases or when significant hemodynamic compromise is present, more intensive immunosuppressive therapies are required, including antithymocyte globulin (ATG) and, historically, anti-CD3 monoclonal antibodies (OKT3). ATG induces lymphocyte depletion through complement-mediated mechanisms and apoptosis. The choice and intensity of treatment are primarily guided by histological severity and the patient’s clinical condition [31,60].

AMR is often associated with a more severe clinical and hemodynamic presentation compared with ACR. Therapeutic strategies include corticosteroids, antibody removal or depletion techniques such as plasmapheresis, and the administration of intravenous immunoglobulin (IVIG). These approaches are frequently combined with B-cell-targeted therapies and plasma cells, including rituximab (anti-CD20 monoclonal antibody) and bortezomib (proteasome inhibitor). In recent years, emerging biological therapies, such as interleukin-6 (IL-6) inhibitors, have also been proposed and investigated, although the quality of the available evidence remains variable. Recent reviews recommend a personalized treatment approach that integrates clinical findings, hemodynamic phenotype, and antibody burden, given the lack of large, well-powered randomized controlled trials for many therapeutic options [61].

Management of CAV is primarily based on strict control of cardiovascular risk factors and targeted pharmacological interventions. Key measures include lipid-lowering therapy, blood pressure control, diabetes management, and smoking cessation. Early initiation of statin therapy has been associated with reduced CAV progression and improved post-transplant mortality, as demonstrated by clinical studies and systematic reviews. Acetylsalicylic acid (ASA) has been proposed to limit platelet activation and slow disease progression, although the available evidence is heterogeneous. Percutaneous revascularization options are often limited by the diffuse and microvascular nature of CAV, while retransplantation remains the only definitive treatment in advanced cases, despite being associated with significant clinical challenges and limited organ availability [62,63].

### 5.3 Management of Chronic Rejection and Long-Term Outcomes

Post-heart transplant outcomes have improved significantly over recent decades, with 1-year survival rates in modern centers approaching 80–90%, although outcomes vary depending on age, indication, and case complexity. Pediatric series and ISHLT reports continue to show improvement in conditional survival curves [64,65].

In the early post-transplant period, acute rejection and perioperative complications (infections, PGD) remain major contributors to mortality. Over the long term, chronic rejection, manifesting primarily as CAV, and immunosuppression-related malignancies are leading causes of late death. Patients who required ECMO or VAD support before transplantation generally have a poorer prognosis [58,64].

Effective management of chronic rejection relies on strict adherence to immunosuppressive therapy and regular surveillance, including laboratory monitoring, DSA screening, and EMB when indicated. Guidelines and observational studies highlight that structured follow-up programs, patient education, and multidisciplinary support enhance compliance and improve long-term clinical outcomes [31,64,66].

### 5.4 Differential Diagnosis of Graft Dysfunction

Early heart transplant failure is not always caused by immune-mediated rejection. Several clinical conditions can mimic acute rejection and should be considered in the differential diagnosis to avoid inappropriate intensification of therapy.

Primary graft dysfunction (PGD) is one of the most serious complications occurring in the immediate post-transplant period. Unlike acute or antibody-mediated cellular rejection, PGD is primarily related to ischemia-reperfusion injury, donor factors, prolonged storage times, and perioperative insults, rather than alloimmune mechanisms. Management is based on intensive hemodynamic support, inotropic drugs and mechanical circulatory support, particularly venoarterial extracorporeal membrane oxygenation (VA-ECMO), which may allow graft recovery in severe cases [58,59].

Other important differential diagnoses include infectious myocarditis, ischemic injury, drug-induced cardiotoxicity, and metabolic disorders, all of which can present with ventricular dysfunction and elevation of biomarkers similar to those of rejection. In these situations, EMB, circulating biomarkers, microbiological investigations, and multimodal imaging are essential to distinguish rejection from non-immune causes of graft dysfunction and to guide appropriate management.

Table 3 (Ref. [31,58,59,60,61]) summarizes the principal therapeutic strategies for heart transplant rejection and other major causes of graft dysfunction.

**Table 3.** **Therapeutic strategies for heart transplant rejection and major causes of graft dysfunction**.

Rejection type/clinical condition	First-line therapy	Adjunctive/second-line therapy	Therapeutic goal
Rejection prevention [31]	CNI (tacrolimus or cyclosporine) + antimetabolite + corticosteroid.	mTOR inhibitors or belatacept.	Maintain immune tolerance with minimal toxicity.
Hyperacute rejection [31]	Urgent antibody removal strategies (plasmapheresis, IVIG) and hemodynamic support.	ECMO and retransplantation in selected cases.	Rapid hemodynamic stabilization.
Acute cellular rejection (ACR) [31,58]	High-dose IV corticosteroids.	ATG, adjustment of maintenance therapy.	Halt myocyte injury and restore function.
Antibody-mediated rejection (AMR) [59]	Corticosteroids + IVIG + plasmapheresis.	Rituximab, bortezomib, eculizumab, tocilizumab.	Antibody removal and immune modulation.
Cardiac allograft vasculopathy (CAV) [60,61]	Statins, ASA, risk factor control.	mTOR inhibitors, revascularization, retransplantation.	Slow disease progression.
Primary graft dysfunction (PGD) [58]	Inotropes and hemodynamic support.	VA-ECMO, temporary VAD, retransplantation in selected cases.	Stabilize graft function and promote myocardial recovery.

CNI, calcineurin inhibitor; ATG, antithymocyte globulin; ECMO, extracorporeal membrane oxygenation; VAD, ventricular assist device; IVIG, intravenous immunoglobulin.

## 6. Arrhythmic Risk and Sudden Cardiac Death Associated With Heart Transplant Rejection

SCD after heart transplantation is a rare but clinically relevant cause of late mortality. Several mechanisms involved in transplant rejection may contribute to the development of an arrhythmogenic substrate [67,68,69,70,71,72].

ACR is characterized by lymphocytic infiltration of the myocardium and myocyte damage, which can alter electrical conduction and increase susceptibility to ventricular arrhythmias. Similarly, AMR induces microvascular endothelial damage and complement activation, promoting myocardial ischemia and electrical instability. Recurrent or subclinical rejection episodes may further contribute to myocardial fibrosis and adverse remodeling [67,68,69,70,71,72].

CAV, the main manifestation of chronic rejection, represents another important mechanism associated with arrhythmic risk. Progressive diffuse coronary involvement and microvascular dysfunction can lead to chronic ischemia, scar formation, and malignant ventricular arrhythmias. These observations suggest that arrhythmic complications should be considered part of the spectrum of graft injury rather than isolated events. Therefore, integrated surveillance strategies combining EMB, DSA assessment, circulating biomarkers, and advanced imaging techniques could improve the identification of patients at increased risk for electrical instability and adverse outcomes [67,68,69,70,71,72].

Although the association between rejection-related inflammation and arrhythmogenesis is increasingly recognized, the precise mechanisms linking immune-mediated damage to ventricular arrhythmias remain to be fully elucidated, and further prospective studies are needed [67,68,69,70,71,72].

## 7. Future Directions and Perspectives

In recent years, cardiac transplantation has seen significant advances in understanding immunological mechanisms and in the development of new diagnostic and therapeutic technologies, paving the way for an increasingly personalized, predictive, and less invasive approach to managing rejection.

The use of advanced molecular biology techniques, such as single-cell transcriptomics (scRNA-seq) and integrated multi-omics analysis, is providing new insights into the immune circuits that regulate cardiac allograft rejection. These technologies allow the identification of alloreactive cell subpopulations, lymphocyte activation states, and early prognostic biomarkers that may overcome the limitations of traditional biopsy [30].

In parallel, noninvasive molecular monitoring methods, such as the quantification of dd-cfDNA and GEP, are demonstrating high sensitivity for the early detection of ACR and AMR, improving risk stratification [50,73].

The most promising prospects concern the personalization of immunosuppressive therapy through biomarker-guided and pharmacogenomic approaches. Recent studies have explored the use of therapeutic drug monitoring of tacrolimus based on intra-lymphocyte concentrations and CYP3A5 genetic variants, as well as the introduction of adaptive regimens with selective reduction of calcineurin inhibitors to minimize renal toxicity while maintaining immunological efficacy. In parallel, the combined use of mTOR inhibitors and new-generation monoclonal antibodies, such as belatacept and anti-CD40L, is the subject of clinical trials to improve immune tolerance and reduce the risk of chronic rejection [74].

A rapidly expanding area of research is cellular and immunological tolerance strategies aimed at reducing or potentially eliminating the need for chronic immunosuppression. Among these approaches, *ex vivo* expanded regulatory T cells (Treg), tolerogenic dendritic cells (tolDC), and mesenchymal stem cell (MSC)-based therapies have shown promising results in preclinical studies and early-phase clinical trials. However, much of the available evidence comes from experimental models or other solid organ transplant settings, particularly kidney transplantation, and these strategies are not currently considered standard of care in heart transplantation. Similarly, gene editing approaches based on CRISPR-Cas9 technology and regulatory CAR-T cells are still largely in the preclinical stage. Although these strategies have demonstrated the potential to induce donor-specific immune tolerance, direct evidence in heart transplantation remains limited and their clinical applicability requires further validation through prospective studies and controlled clinical trials [75,76,77].

The EMB remains the primary diagnostic reference, but the need to reduce its invasiveness and advance diagnosis has pushed towards multimodal surveillance protocols. The integration of plasma biomarkers, advanced echocardiographic techniques (including tissue Doppler imaging and speckle tracking strain analysis), cardiac MRI with T1/T2 mapping, immunological PET, and artificial intelligence algorithms applied to clinical and molecular data represents one of the most promising prospects for the future. The goal is to move from a reactive model to a proactive and predictive approach, capable of identifying at-risk patients early and guiding the modulation of immunosuppression in real time, improving the survival and quality of life of transplant patients [53,78,79,80].

Digital pathology and artificial intelligence are emerging as powerful tools in transplant pathology. Machine learning algorithms applied to whole-slide images of endomyocardial biopsies may improve reproducibility, reduce interobserver variability, and enable the identification of subtle rejection patterns. When integrated with molecular and clinical data, these approaches support a truly integrated diagnostic model.

Integrated diagnosis, combining histology, molecular profiling, circulating biomarkers, and clinical parameters, represents a cornerstone of precision medicine. This multimodal approach enables personalized risk stratification and dynamic adjustment of immunosuppressive therapy (Fig. 3).

**Fig. 3.**
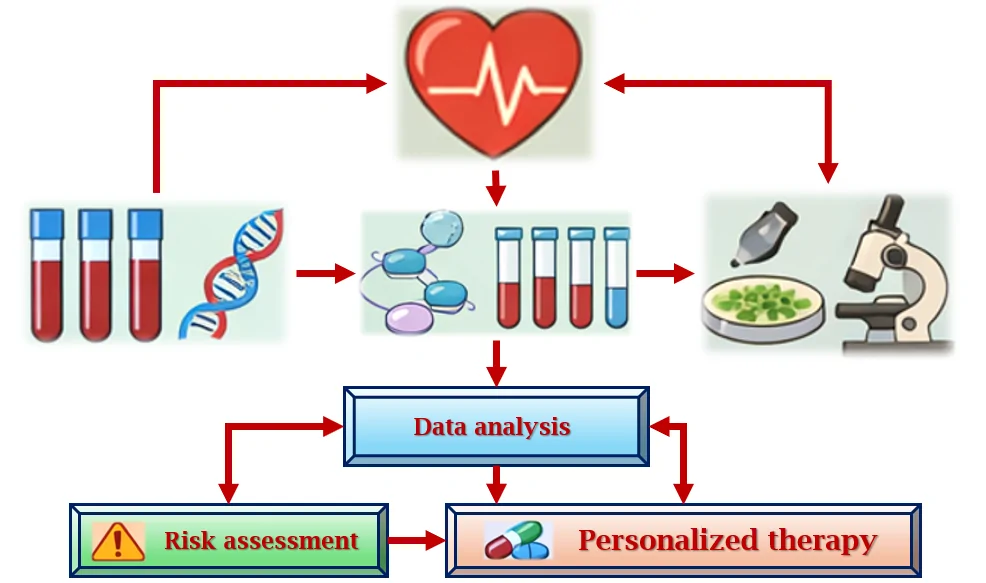
**Precision medicine in heart transplant monitoring**.

## 8. Conclusions

HTR remains one of the major challenges in contemporary cardiovascular medicine. Despite significant advances in transplant surgery and immunosuppressive protocols, the recipient’s immune response to the allograft continues to account for a substantial proportion of both short- and long-term morbidity and mortality. A key objective of modern transplant medicine is the transition from a reactive diagnostic approach, based primarily on histopathological evidence of injury, to predictive and personalized surveillance strategies capable of identifying rejection before overt graft dysfunction develops.

The role of the cardiovascular pathologist remains central to the diagnostic process. EMB is still the gold standard for the histological evaluation of rejection, allowing for the identification of cellular and humoral patterns and staging according to ISHLT criteria. However, the evolution towards molecular and non-invasive methods, such as the measurement of donor-derived cell-free DNA, GEP and advanced immunophenotyping, is redefining the boundaries of traditional histopathological diagnosis, integrating it into a broader perspective of precision medicine.

Among noninvasive diagnostic tools, advanced echocardiography has established itself as a key element of long-term graft surveillance, allowing real-time assessment of myocardial mechanics and early identification of subclinical dysfunction. Together with molecular biomarkers and histopathological assessment, echocardiography contributes to a multimodal and personalized approach to monitoring rejection.

The integration of morphology, molecular biology, and immunopathology will improve the predictive capacity of diagnostic tests and personalize the therapeutic approach, minimizing subclinical rejection events and complications related to chronic immunosuppression. In this context, the pathologist plays a key role in linking the laboratory and clinical practice, contributing to the definition of multimodal diagnostic strategies and dynamic surveillance protocols.

Future prospects lie in the development of integrated diagnostic platforms, the application of artificial intelligence to histomorphometric analysis, and the progressive transition towards models of guided immunological tolerance. Only through a multidisciplinary synergy between pathologists, immunologists, and cardiologists will it be possible to transform the management of rejection from a reactive process to a predictive paradigm, improving the prognosis and quality of life of transplant patients.
